# Endothelial Dysfunction and Diabetic Cardiomyopathy

**DOI:** 10.3389/fendo.2022.851941

**Published:** 2022-04-07

**Authors:** Moran Wang, Yongsheng Li, Sheng Li, Jiagao Lv

**Affiliations:** ^1^ Division of Cardiology, Department of Internal Medicine, Tongji Hospital, Tongji Medical College, Huazhong University of Science and Technology, Wuhan, China; ^2^ Department of Emergency, Tongji Hospital, Tongji Medical College, Science and Technology, Huazhong University, Wuhan, China

**Keywords:** diabetic cardiomyopathy, endothelial dysfunction, oxidative stress, metabolism, diabetes

## Abstract

The cardiovascular complications contribute to a majority of diabetes associated morbidity and mortality, accounting for 44% of death in those patients with type 1 diabetes mellitus (DM) and 52% of deaths in type 2 DM. Diabetes elicits cardiovascular dysfunction through 2 major mechanisms: ischemic and non-ischemic. Non-ischemic injury is usually under-recognized although common in DM patients, and also a pathogenic factor of heart failure in those diabetic individuals complicated with ischemic heart disease. Diabetic cardiomyopathy (DCM) is defined as a heart disease in which the myocardium is structurally and functionally abnormal in the absence of coronary artery disease, hypertensive, valvular, or congenital heart disorders in diabetic patients, theoretically caused by non-ischemic injury solely. Current therapeutic strategies targeting DCM mainly address the increased blood glucose levels, however, the effects on heart function are disappointed. Accumulating data indicate endothelial dysfunction plays a critical role in the initiation and development of DCM. Hyperglycemia, hyperinsulinemia, and insulin resistance cause the damages of endothelial function, including barrier dysfunction, impaired nitric oxide (NO) activity, excessive reactive oxygen species (ROS) production, oxidative stress, and inflammatory dysregulation. In turn, endothelial dysfunction promotes impaired myocardial metabolism, intracellular Ca^2+^ mishandling, endoplasmic reticulum (ER) stress, mitochondrial defect, accumulation of advanced glycation end products, and extracellular matrix (ECM) deposit, leads to cardiac stiffness, fibrosis, and remodeling, eventually results in cardiac diastolic dysfunction, systolic dysfunction, and heart failure. While endothelial dysfunction is closely related to cardiac dysfunction and heart failure seen in DCM, clinical strategies for restoring endothelial function are still missing. This review summarizes the timely findings related to the effects of endothelial dysfunction on the disorder of myocardium as well as cardiac function, provides mechanical insights in pathogenesis and pathophysiology of DCM developing, and highlights potential therapeutic targets.

## Introduction

It was estimated that the global diabetes prevalence would rise to 10.2% (578 million) in 2030 (1). The prediction from the World Health Organization is that diabetes will be the seventh leading cause of death by 2030. Diabetes severely endangers public health, especially its cardiovascular complications, which are the largest challenge accounting for 50%-80% of death of diabetic patients (2). HbA1c, the important index to evaluate blood glucose levels, is not sufficient to predict the risk of macrovascular events (3, 4). Therapeutic management and monitoring of blood glucose are the basis of diabetes therapy. However, present therapeutic strategies do not reduce the fatality rate of cardiovascular complications in diabetes. This finding suggests that metabolic disturbance is not the exclusive cause for macrovascular events followed by diabetes and other metabolic disorders, highlighting the contribution of other factors. Diabetic cardiomyopathy is an outcome of intrinsic heart muscle malfunction, different from atherosclerotic vascular disease (5, 6). The etiology of diabetic cardiomyopathy is complex, with a change in cardiomyocyte metabolism, which has been considered as a pivot stimulus for cardiomyopathy dysfunction (7).

Diabetic cardiomyopathy is a significant entity characterized by diastolic impairment and left ventricular hypertrophy in the absence of vascular defects (7, 8). Furthermore, it has been identified as a microvascular complication, in which cardiac microvascular endothelial dysfunction in the first ring of diabetic cardiomyopathy and running throughout the entire process, suggested by many studies (9, 10). The development of diabetic cardiomyopathy has been reported correlated with several factors, including decreased cardiac compliance, insulin resistance (11, 12), endothelial dysfunction, increased oxidative stress (11), aberrant ion flux (13), and coronary microcirculation abnormalities (12). It has been widely documented that endothelial dysfunction occurs in diabetes patients and in individuals with insulin resistance or at high risk for developing type 2 diabetes.

Therapeutic methods targeting specific molecules modification in microvascular endothelial cells (MVECs) and cardiomyocytes exposed to high glucose stimuli have shown beneficial effects in clinical trials (14, 15). Hyperglycemia has been identified as an important contributor to endothelial dysfunction in both type 1 and type 2 diabetes (16, 17). Microvascular endothelial dysfunction is primarily characterized by decreased release of NO, enhanced oxidative stress, increased production of inflammatory factors, abnormal angiogenesis, and impaired endothelial repair (18). Endothelial cells and cardiomyocytes both are major components of the heart, and their interaction is complicated and crucial in the progression of diabetic cardiomyopathy. Cheang et al. reported endothelial nitric oxide synthase enhancer increased NO bioavailability and reduced oxidative stress, further ameliorating endothelial dysfunction in db/db mice (19).

The endothelium is the biggest organ of the body, maintains and regulates the normal function of vessels. The endothelium functions as a mechanical lining in vessels, it also plays a pivot role in the regulation of leucocyte-adhesion, platelet aggravation, and blood vessel patency. Endothelium functions by regulating the release of secretory factors in response to mechanical stimuli. The major role of endothelium is to ensure adequate blood flow, which is dependent on the counterbalance between vasodilators and vasoconstrictors. Vasodilators, including prostacyclin I2 (PGI2) and nitric oxide (NO), aim to maintain adequate blood through dilating the vessels; vasoconstrictors, including endothelin-1 (ET1) and thromboxane A2 (TXA2), address to counterbalance the excessive vasodilation and maintain the vascular tone. Insulin resistance and diabetes have both been reported initiated or associated with endothelial dysfunction. In addition, endothelial dysfunction is correlated with obesity, sedentary lifestyle, and smoking. These observations suggest the complex pathophysiology of endothelial dysfunction involves multiple mechanisms and take part in various diseases. Therefore, we will discuss the role of endothelial dysfunction in the development of diabetic cardiomyopathy in followed sections.

## Endothelial Dysfunction in Diabetes

### Altered Vascular Endothelial Barrier Function

Chronic hyperglycemia results in metabolic derangements in endothelial cells and organs damage. It has been indicated that hyperglycemic conditions not only impair endothelial cells by increasing ROS and inhibiting NO synthase, but also promote increased permeability of the endothelial cells layer (20). The activation of diacylglycerol (DAG) - Protein Kinase C (PKC) signaling pathway contributes to the increased permeability of endothelial cells (21, 22). It was reported that the activation of cPKC and nPKC is DAG dependent, and associated with increased vascular permeability and leukocyte adhesion in diabetes, involving the heart and kidney (23). In human umbilical vein endothelial cells (HUVECs), PKC leads to the phosphorylation of the myosin light chain (MLC), further inducing phosphorylation modification of VE-cadherin on tyrosine and the disruption of adherence junctions (24). There are several protein families taking part in the formation and regulating tight junction in endothelial cells, including transmembrane, scaffolding, and signaling proteins (25). Diabetic condition induced decreased expression of occluding elevated contents of glycated-occludin, and the loss of transendothelial electrical resistance (TEER) in endothelial cells. The disruption of the endothelial barrier is closely associated with excessive oxidative stress, N-acetylcysteine (NAC) prevented endothelial cell dysfunction induced by high glucose exposure (26). The precise mechanisms of regulating these proteins are still uncertain and deserved further investigation. In addition, it has been well documented that oxidative stress impaired endothelial barrier function *via* promoting Ca^2+^ influx into endothelial cells to disrupt inter-endothelial tight junctions. Overactivated TRPM2-mediated Ca2+ signaling leads to the internalization of VE-Cadherin and degradation of ZO-1, further increasing trans-endothelial migration of neutrophils in response to various pathological factors induced by ROS (27).

### Abnormal Nitric Oxide Synthase Activity

Impaired NO signaling is closely correlated with myocardial damage in diabetes mellitus, characterized by the dysregulation of NO generation and bioavailability (28). Felaco et al. (29) reported the reduced deposition of eNOS in endothelial cells in diabetic rat hearts, compared with non-diabetic controls, without influence on the protein level or the mRNA level of eNOS in the heart. This observation highlights the correlation between the diabetes pathogenesis and expression of eNOS in cardiac ECs of diabetic rats, suggesting the important role of cardiac ECs. Further studies reported by Sampaio et al. (30) indicated the cardiac abnormalities observed in diabetic rats similar to the counterparts in L-NAME-induced cardiomyopathy, suggesting the pivot role of NO in the pathophysiology of DCM. In addition, inhibition of NOS reduced the levels of NO, nitrotyrosine, and reactive oxygen species (ROS), indicating eNOS uncoupling in diabetic hearts (31, 32). The oxidation of tetrahydrobiopterin induced by reactive oxygen species (ROS) and increased content of asymmetric dimethylarginine (ADMA) contribute to eNOS uncoupling in DM.

iNOS is an inductive isoform of NOS, whose expression increases while addressing cytokines and other agents (33). The altered expression and activity of iNOS have been reported to contribute to diabetes-associated cardiovascular complications. The expression of iNOS has been reported to increase in mesenteric arteries and hearts of diabetic rats (34–36). Puthanveetil et al. (37) reported iNOS caused cardiomyocyte cell death by mediating the nitrosylation of GAPDH and caspase-3. Excessive iNOS formation causes contractile dysfunction and heart failure when iNOS uncouples, produces ROS, and contributes to oxidative stress due to limited substrates. Moreover, oral sepiapterin improved left ventricular function in diabetic mice by inhibiting iNOS uncoupling (38).

Besides eNOS and iNOS, there are some studies about the role of nNOS in diabetic cardiomyopathy. However, the alterations of iNOS expression are still controversial (36, 39, 40). Previous studies suggested that PKC-β_2_ induced iNOS upregulation in DCM may be mediated by RhoA/ROCK pathway. And a recent study by Lei et al. reported that the inhibition of PKC-β_2_ caused increased expression of caveolin-3, augmented the phosphorylation of eNOS, and decreased iNOS expression, further improving diastolic dysfunction in diabetic rats. In addition, asymmetric dimethylarginine (ADMA), an endogenous inhibitor of NO synthase, has elevated in the plasma of patients with type 2 diabetes (41). Further studies suggest that hyperglycemia induces the impaired activity of dimethylarginine dimethylaminohydrolase (DDAH), leading to ADMA accumulation and may cause a reduction of NO expression and endothelial vasodilator dysfunction in diabetes (42). Moreover, AGEs quench nitric oxide activity *in vitro* and *in vivo* and mediate impaired vasodilation (43).

### Reactive Oxygen Species (ROS) Generation and Oxidative Stress

It has been widely reported oxidative stress resulting from an imbalance between pro-oxidative and anti-oxidative compounds, significantly augments in long-standing diabetic cardiomyopathy (44, 45). Increased glucose flux through activating NAD(P)H oxidase (NOX) and following augmented the superoxide anion (O_2_^-^) production results in the production of oxidative stress (46). Increased O_2_^-^ anion acts with hydroxyl radicals and hydrogen peroxide, which may lead to oxidant injury (47). Superoxide may react with NO to produce peroxynitrite (ONOO^−^), further leading to an increase of lipid peroxidation, protein nitration, and oxidizing low-density lipoproteins (LDLs) (48). Peroxynitrite causes eNOS uncoupling by oxidation of BH_4_ and oxidation of the zinc-thiolate center of eNOS (49, 50). In addition, excessive ADMA contributes to an imbalance between NO and ROS, *via* stimulating superoxide release from endothelial cells (51).

Abnormal metabolism and dysfunction of mitochondria are related to aberrant metabolism in diabetic cardiomyopathy. It has been reported that hyperglycemia causes oxidative damage of mitochondrial DNA in endothelial cells, by increasing mitochondrial reactive oxygen species (ROS) (52). Damaged mitochondria DNA activates the (poly ADP-ribose) PARP-1 pathway in the nucleus of endothelial cells, further leading to inhibition of Glyceraldehyde-3-Phosphate Dehydrogenase (GAPDH), further impaired glycolysis process The inhibition of glycolysis causes the accumulation of glycolytic intermediates, resulting in relatively enhanced branching pathways of glucose metabolism including the polyol pathway, the hexosamine biosynthesis pathway, or the glycation pathway (53). Importantly, these pathways all contribute to endothelial dysfunction. Further experimental evidence suggested that inhibition of PARP-1 in endothelial cells prevent endothelial dysfunction induced by diabetes (54).

Other mechanisms are involved in endothelial dysfunction induced by excessive ROS exposed to high glucose. It was reported that induction of sphingosine-1-phosphate receptor 1 (S1PR1) or reduction in S1PR2 both improve endothelial dysfunction (55). In addition, activation of AMPK shows an inhibitory effect on overproduction of mitochondrial ROS (mtROS) in endothelial cells. And it has been shown the upregulation of AMPK may prevent endothelial dysfunction in diabetic mice (56). Taken together, these findings suggest the important role of endothelial dysfunction in diabetic cardiomyopathy, and ROS inhibition may be a therapeutic target for diabetic cardiomyopathy.

### Inflammation

To address damage factors, endothelial cells are activated and produce interleukins, chemokines, interferons, monocyte chemoattractant protein-1 (MCP-1), and other inflammatory factors (57). Monocytes and neutrophils are recruited to the activated endothelium following the release of these substances, and initiate inflammation. Elevated proinflammatory factors stimulate endothelial cells to secrete other proinflammatory factors, which induce the secretion of diverse acute-phase reactants and modulate chronic inflammation. It has been reported that insulin inhibits NO bioavailability *via* the p38 MAPK-cFOS pathway and increases inflammation in hyperinsulinemic insulin-resistant subjects. Furthermore, the inflammatory levels are elevated when in endothelial cells exposed to hyperinsulinemic serum. In addition, mitochondrial dysfunction induces endothelial dysfunction and promotes inflammation by producing excessive ROS (58, 59). The inflammatory state triggered by endothelial activation may be the consequence of an imbalance of excessive ROS and insufficient antioxidants, resulting in oxidative stress and cell damage.

### Hemodynamic Alterations

Vascular endothelial cells are prone to be damaged by hyperglycemia owing to its characteristic and location. Damaged endothelial cells cause permeability increase, barrier dysfunction, and vasodilatation impairment (10, 46, 60). Many studies on hemodynamic studies all suggested that endothelium-dependent vasodilatory response in diabetes is impaired (61, 62). The balance between the vasoconstrictors and vasodilators is impaired, which are released by endothelial cells to help maintain coronary vascular structure and normal blood flow. Diabetic cardiomyopathy causes the over-release of various vasoconstrictors.

These alterations are associated with increased vasoconstriction and impaired vasodilatation, caused by aberrant levels of vasodilators, with NO and PGI2 as representatives, as well as vasoconstrictors, represented by ET-1 and TAX2. Moreover, ET-1 is upregulated in the target organs of diabetic complications, including the heart, and kidney (63, 64). It was reported that ET-1 is predominantly expressed in cardiac endothelial cells compared with cardiomyocytes in normal adult heart tissue, highlighting the important role of endothelial cells in diabetic cardiomyopathy (65). Moreover, increased endothelin production in the diabetic heart may lead to vessel hypertrophy and increased myocardial fibrosis, which both are the characteristic of diabetic cardiomyopathy (66, 67). Excessive vasoconstrictors prostanoids increased ROS production by upregulating NADPH oxidase and type 4 and type 5 phosphodiesterases (PDE4 and PDE5) (67, 68).

A crucial component involved in endothelium-dependent relaxation is NO. NO is synthesized by nitric oxide synthase (NOS) catalyzed by L-arginine and NADPH. in the presence of oxygen. However, in diabetic vessels, NO synthesis damages. Some studies suggested that it may be the inactivation of NO that causes NO deficiency due to an increase in free radicals, instead of the downregulation of activity or expression of eNOS (66, 69, 70).

### Aberrant Endothelial Cell Metabolism

In ECs, GLUT1 is the major isoform among diverse glucose transporters. In the view of the insulin-independent characteristic of GLUT1 and the “first response” location of ECs while in the condition of hyperglycemia, ECs are susceptible to high blood glucose. The expression of GLUT1 was previously thought of as unresponsive to hyperglycemia (71, 72). However, some studies indicated that the expression of GLUT-1 and the rate of glucose transport is downregulated, in response to extended exposure to high glucose concentrations (73). Thioredoxin-interacting protein (TXNIP), which is upregulated in response to low insulin and HG, reduces the level of GLUT1 mRNA (74, 75). These adaptive changes of GLUT1 expression may protect ECs against the damage of excessive glucose influx by reducing the uptake of glucose. On the other hand, the downregulation of GLUT1 would lead to decreased glucose efflux across the abluminal border, further reducing glucose expulsion to the cardiomyocyte. Hypoxic and inflammatory conditions occurring in diabetic cardiomyopathy promote endothelial cells to revascularizing tissue to address scarce oxygen and nutrients. During the period of vessel sprouting and migration, the glycolytic flux is enhanced (76). Enhanced glycolytic flux facilitates endothelial cells to migrate into the hypoxic area and proliferate in metabolism-impaired cardiac tissue.

In addition, insulin-independent glucose uptake of GLUT1 in endothelial cells results in an increase of glucose concentrations in endothelial cells. Excessive glucose in endothelial cells would be shunted into side branches of glycolysis, such as the pentose phosphate pathway, the hexosamine biosynthesis pathway, the polyol pathway, and the glycation pathway, leading to angiogenesis impairment, mitochondrial dysfunction, and protein kinase C activation (77). These alterations ultimately cause excess ROS and reactive nitrogen species production, and advanced glycation end product (AGE) synthesis, ECs, furthermore the impaired function of cardiomyocytes (71, 77–79). During diabetes, excessive AGEs cause ECs dysfunction by binding to the receptor for advanced glycation end products (RAGE), resulting in endothelial cell permeability increase (80), eNOS activity inhibition (81), and the coagulation system impairment (82, 83), NADPH oxidative (NOX) and NF-kB activating (84–86).

## Pathophysiologic Alteration of Cardiomyocytes

Diabetic cardiomyopathy has two phenotypes: hyperglycemia, lipotoxicity, and insulin resistance are more important factors for DCM with HFpEF phenotype, autoimmunity is particularly related with DCM with HFrEF phenotype, and AGEs deposition and microvascular rarefaction seem to contribute to both phenotypes (87). Type 2 diabetes mellitus (T2DM) comprises 90–95% of all people with diabetes. Mounting evidence points to endothelial dysfunction of the coronary microvessels as a pivotal contributor to restrictive left ventricular (LV) remodeling and diastolic dysfunction, and subsequent heart failure with preserved ejection fraction (HFpEF), the most common form of HF in DM (88, 89). DM-related metabolic derangements favor the development of the HFpEF phenotype, which is more prevalent in obese type 2 DM patients.

Endothelial cells are the third most abundant cell type in the mammalian heart, accounting for about 12% of atrial and 8% of ventricular cell numbers respectively (90). The cardiac vasculature consists of abundant capillaries for the large requirement of cardiac muscle which is the most aerobic organ in the body. The ratio of muscle to capillary in the heart is about 1:1, and the distance between a capillary endothelial cell and a neighboring close cardiomyocyte is approximately 1 μm, suggesting the intimate relationship and intercellular dependence between endothelial cells and cardiomyocytes. Endothelial cell is a critical component in cardiac tissue, which regulates cardiac constriction and blood flow which provides oxygen and nutrients for cardiomyocytes. Impaired NO bioactivity and oxidation of BH4 which functions as a NOS cofactor, have been well documented in diabetes and diabetes-associated complications. Recently, Carnicer et al. reported that increased concentration of BH4 in cardiomyocytes prevented and attenuated LV dysfunction through improving NO activity and further leading to enhance insulin-independent glucose uptake and utilization. Importantly, there is experimental evidence supporting that endocardial and endothelium regulate cardiomyocyte development and maturation by secreting endocrine.

It has been well documented that endothelial-derived cardio-active factors including nitric oxide (NO), endothelin-1, neuregulin-1(NRG-1), and Prostaglandin I2, regulate cardiomyocyte activity. Recently angiopoietins, angiotensin II, prostaglandins, connective tissue growth factor, fibroblast growth factor, vascular endothelial growth factor, Dickkopf-3, apelin, and endothelial miRNAs have been added into the panel of endothelial-derived cardio-active factors (91).. A large amount of endothelial-derived cardio-active factors and the specific modulation of each one emphasizes the precise regulation net of endothelial cells on cardiomyocytes. Cardiomyocyte aberrant metabolism and coronary microvascular dysfunction (CMD) are major pathogenesis of diabetic cardiomyopathy. Coronary microvascular dysfunction is characterized by endothelial cell damage, closely correlated with the incidence of heart failure in diabetic patients.

### Regulation on Cardiomyocyte Metabolism

Heparanase secreted from endothelial cells binds Heparan sulfate proteoglycans (HSPGs) which are located in the membrane of cardiomyocytes, triggering the release of LPL and VEGF from cardiomyocytes. LPL is released onto HSPG-binding sites on the plasma membrane, then captured by glycosylphosphatidylinositol-anchored high-density lipoprotein binding protein 1 (GPIHBP1) and transferred across to the vascular lumen. In hyperglycemia conditions, above-mentioned process is intensified, resulting in a rapid increase of LPL and FA at the vascular lumen. This process provides increased FA for diabetic cardiomyocytes to generate ATP and maintain normal function as a compromised method to address acutely diabetic cardiomyopathy. Abnormal cardiac mitochondrial metabolism causes decreased ATP synthesis and a lower myocardial creatine phosphate/ATP ratio.

### Mitochondrial Defect

Mitochondrial dysfunction promotes the development of diabetic cardiomyopathy. Excessive FFA, exceeding the capacity of mitochondrial β-oxidation, promotes the accumulation of toxic metabolic intermediates and further mitochondrial dysfunction and cell death. This process is referred to as lipotoxicity and has been reported in numerous DM animal models. Moreover, Excessive mitochondrial fatty acid uptake and β-oxidation cause a large number of consumptions of ATP, resulting in mitochondrial dysfunction. During the advanced stage of diabetic cardiomyopathy, mitochondrial biogenesis and respiratory function is impaired severely owing to the abnormality of adenosine monophosphate-activated protein kinase (AMPK) signaling pathway, leading to mitochondrial dysfunction (11). Intracellular Ca^2+^ mishandling further results in mitochondrial respiratory dysfunction leading to cell death. Opening of the mitochondrial permeability transition pores induced by overload Ca^2+^ results in cardiomyocyte autophagy and cardiac necrosis. Impaired mitochondrial function further exacerbates aberrant redox imbalance and leads to reduction of mitochondrial calcium concentration [mito-(Ca^2+^)]. Experimental evidence indicated increased mito-Ca^2+^ uptake by pharmacological or genetic facilitation, improving cardiac systolic dysfunction. Reticulum-mitochondria Ca^2+^ miscoupling disrupted mitochondrial bioenergetics and organelle Ca^2+^ exchange, further damaging cell contraction (92). On the other hand, sustaining lipid accumulation and mitochondrial dysfunction induces ROS generation, which in turn leads to further impaired mitochondrial function and decreased FAO capacity, resulting in lipid accumulation, diastolic dysfunction and eventually heart failure.

### Intracellular Ca^2+^ Mishandling and Endoplasmic Reticulum Stress

In DCM, impaired insulin signaling results in decreased glucose uptake into cardiac myocytes and activity of efflux pumps, leading to the increase of intracellular calcium and subsequently affecting the dynamic balance of the contraction-relaxation cycle of the cardiomyocytes (11). In diabetes patients, abnormal insulin metabolic signaling causes the production of reactive oxygen species (ROS) and subsequently induces oxidative damage. Oxidative damage impairs the function of RyR, ATPase pumps and exchange channels. The depression of insulin-stimulated coronary endothelial nitric oxide synthase (eNOS) activity and increased NO production, are the results of abnormal insulin metabolic signaling, leading to the decrease of Ca^2+^ sensitization and reducing sarcoplasmic Ca^2+^ uptake in cardiomyocytes.

Cardiac oxidative stress, lipotoxicity, inflammation, and the accumulation of misfolded proteins lead to cardiac endoplasmic reticulum (ER) dysfunction, induce the unfolded protein response (UPR) and promote ER stress. ER stress and the unfolded protein response led to decreased cellular protein synthesis and accumulation of damaged proteins, ultimately inducing cell apoptosis and autophagy. Increased apoptosis further endangers diabetic cardiomyopathy. It was reported that diabetic cardiac tissue showed cardiomyocyte apoptosis 85-fold increase than the counterpart in control hearts. ER stress also induces autophagy through a Ca^2+^-dependent pathway. The activation of autophagic response is compensatory feedback to protect the cell from apoptosis (8). Autophagy function is impaired in diabetic cardiomyopathy (93). Yang et al. reported inflammation to induce UPR dysfunction through iNOS-mediated S-nitrosylation of IRE1α, which causes impaired IRE1α activity, ER dysfunction, and prolonged ER stress in obesity (94). Zhou and colleagues showed S-nitroso-coenzyme A (SNO-CoA) delivers nitric oxide to the enzyme pyruvate kinase M2 (PKM2), which is modified with S-nitrosylation, thereby inhibiting glycolysis (95). Glucose turns to produce NADPH, a cofactor used by antioxidants, further generates the antioxidants to inhibit excessive oxidative stress (95). Growing evidence indicates the great influence of S-nitrosylation in UPR and cellular metabolism, suggesting the pivot role of nitric oxide signaling pathway and endothelial function in metabolic disorders. Abnormal endothelial function induces the imbalance between components derived from endothelial cells. ET-1 stimulates the entry of extracellular Ca^2+^ and activates the intracellular PLC/IP3/Ca^2+^ pathway *via* cGMP-dependent pathway (92).

### Accumulation of Advanced Glycation End Products

Abundant myocardial microvascular AGEs deposition in diabetic cardiomyopathy has been reported by mounting evidence. Further, the deposition of AGEs triggers vascular inflammation and quenches endothelially produced NO, resulting in decreased myocardial NO bioavailability and the predisposition to restrictive LV remodeling. AGEs deposition also occurs in the myocardial interstitium between cardiomyocytes. Interstitial AGEs deposition triggers ROS production in cardiomyocytes by NADPH oxidase, further leading to the activation of cell death pathways and eccentric LV remodeling (96). Hyperglycemia induces a protein glycation reaction of non-enzymatic glycosylation of lipids, lipoproteins, and amino acids, leading to the increase of AGEs. Elevated AGE deposition leads to increased connective tissue crosslinking, fibrosis, cardiac stiffness, thereby impairing diastolic relaxation. A RAGE antagonist ameliorated myocardial collagen deposition, fibrosis, stiffness, and diastolic dysfunction (97).

### ECM Deposition

Diabetic cardiomyopathy is characterized by cardiac interstitial and perivascular fibrosis, which contribute to the development of diastolic dysfunction correlated with a high prevalence of heart failure with preserved ejection fraction (HFpEF) in patients with diabetes. The pathogenesis of diastolic dysfunction is closely related to extracellular matrix (ECM) deposition. Sustaining hyperglycemia damaged endothelial cells, ultimately leading to cell loss and reduced coronary microcirculation blood flow (98). In addition, elevated factors including transforming growth factor-beta (TGF-β) and ET-1 both promote the expression of fibronectin, playing an important role in regulating ECM composition (99). In the diabetic hearts of rats, the expression of fibronectin and collagen IV are increased. Interestingly, Bosentan, an ET-1 receptor antagonist, exhibits prevention the increased expression of fibronectin and collagen IV in diabetic hearts of rats (66). Moreover, ET-mediated BM thickening and myocardial fibrosis resemble those in diabetic rats (100).

Sustained activation of inflammatory pathways ultimately would lead to excessive deposition of ECM. In the period of ECM accumulation, myofibroblasts are the crucial mediators (101). In normal conditions, myofibroblasts are usually removed by apoptosis at the end of the repair. In pathological situations, ECM deposition occurs under the uncontrolled activation of myofibroblasts. Endothelial cells transform to fibroblasts, through a process known as endothelial to mesenchymal transition (EndMT), which is triggered by high glucose concentrations, inflammation, and vascular complications (102, 103). It was thought that EndMT is the key link between inflammation and endothelial dysfunction in diabetic complications (104). In the process of EndMT, endothelial cells lose their typical cobblestone morphology, tight junctions, and typical markers, they acquire increased motility, secretion function of ECM proteins, and begin to express several mesenchymal markers (105). Several pathways are involved in EndMT regulation, including transforming growth factor-beta (TGF-β) signaling, Notch signaling, fibroblast growth factor/fibroblast growth factor receptor 1 (FGF/FGFR1) signaling pathway, Smad2/3-mediated pathways, Wnt-β/Catenin pathway, and pro-inflammatory signaling cascades (106, 107). Single-cell RNA sequencing data revealed that chronic exposure to high glucose and inflammation initiates ECM deposition, eventually perpetuating TGF-β signaling and EndMT (108).

ECM deposition and cardiac fibrosis increase the distance between capillaries and myocytes, leading to oxygen diffusion slowing down and exposing the myocardium to the risk of hypoxia. The expression of VEGF and its receptor have been shown downregulated in cardiac tissues of diabetic animals and humans (109). This change in the heart would further exacerbate hypoxic conditions and result in severe damage. Angiogenesis increases blood flow and reduces vasoconstriction, which may be an important therapeutic intervention for diabetic cardiac tissues. Moreover, VEGF gene therapy in animal studies has shown amelioration in diabetic cardiomyopathy (44). In the future, therapeutic administration of VEGF in clinical trials targeting diabetic cardiomyopathy would provide further evidence.

### Left Ventricle Remodeling and Cardiac Stiffness

Diabetes mellitus leads to Left ventricle (LV) diastolic dysfunction, restrictive LV remodeling, and HFpEF, by inducing a proinflammatory state accompanied by impaired coronary microvessel endothelial function. In HFpEF patients, coronary microvascular endothelial inflammation and cardiomyocytes exposed to altered paracrine endothelial signaling mainly contribute to concentric LV remodeling (87). The association between depressed NO bioavailability in coronary microvessels and impaired cardiac diastolic function is complex, involving multiple pathways. Troponin I and titin protein kinase G-dependent hypophosphorylation is responsible for delayed LV active relaxation and lower passive LV distensibility. Depressed coronary flow reserve (CFR) is also an important contributor. Microvascular endothelial dysfunction leads to impaired NO-dependent braking addressing pro-hypertrophic stimuli, resulting in LV hypertrophy with enlarged cardiomyocytes (110). In addition, abnormal endothelial function is accompanied by increased adhesion molecules and local infiltration, inducing the transformation of myocardial fibroblasts into myofibroblasts and the consequent occurrence of reactive interstitial fibrosis. The HFpEF phenotype of DCM showed worse clinical manifestations, with more frequent hospitalizations and less exercise capacity, characterized by LV hypertrophy and higher LV stiffness. Previous studies reported that these hemodynamic features attributed to microvascular advanced glycation end-products (AGEs) deposition and stiff cardiomyocytes.

## The Regulation of Endothelial Cells on Cardiomyocytes

Similar to the vascular endothelium regulating vascular smooth muscle contraction addressing to shear stress of flowing blood, the contractile state of cardiomyocytes is regulated by the endocardial endothelial cells and the endothelial cells of intramyocardial capillaries. Endothelial cells within the heart regulate myocardial contractile function by releasing bioactive substances, including nitric oxide, endothelin, prostanoids, adenylpurines, and other agents. Exosomes derived from ECs may regulate cardiac remolding in DCM and the development of DCM. The precise regulation of cardiomyocytes by endothelial exosomes needs further investigation, exosome cargoes delivering siRNAs and drugs is a potential strategy in diabetic cardiomyopathy (111). These agents mainly function by modifying the properties of cardiac myofilament, rather than altering cytosolic Ca^2+^ transients, thus leading to different modulation effects on myocardial relaxation and diastolic tone (112). Yao et al (113). demonstrated in endothelial cells hyperglycemia induces a series of changes, which are all memorized by endothelial cells and not erased when switched to a low glucose condition, leading to perivascular fibrosis and cardiac dysfunction. Moreover, the production of NO, generation of ROS, and the mitochondrial oxygen consumption rate are found similar to the metabolic memory in endothelial cells. *In vivo*, the disruption of endothelial cell metabolic memory restores cardiovascular function by regulating corresponding signaling pathways in diabetic mice, whereas insulin alone does not improve cardiac function (113). This regulation on cardiac myocyte function by paracrine of endothelial cells is worth investigating both physiologically and in pathological states (112).

### ET1

Endothelin (ET)-1 is an amino acid peptide with vasoconstrictive effects, which is produced and released by endothelial cells. ET1 binds to ETB receptors on cardiac endothelial cells, triggering the release of NO and PGI2, rather than vasoconstriction. While binding to the ETA and ETB receptors on cardiomyocytes, Gq protein is activated which increases the release of sarcoplasmic reticulum calcium, resulting in the contraction of cardiomyocytes. Moreover, it has been reported the activation of ETA and ETB receptors on cardiomyocytes is associated with the development of cardiac myocyte hypertrophy. Importantly, Zhao et al. demonstrated that endogenous endothelin-1 is necessary to maintain normal cardiac function and cardiomyocyte survival in mice and ET1 upregulates NF-kappaB signaling to diminish TNF-related apoptosis (114).

### NO

Coronary microvessel-derived NO activates soluble guanylate cyclases, further promoting the production of cyclic guanosine monophosphate, which regulates the onset of ventricular relaxation and maintains normal pump function. Impaired NO availability results in low cGMP levels and decreased PKG activity, leading to hypo-phosphorylation of titin and an increase in cardiomyocyte stiffness (115).

### Prostaglandin I2

PGI2 is a physiologically active lipid compound synthesized from arachidonic acid. PGI2 has been reported to regulate cardiomyocyte morphology and survival. Treatment with PGI2 demonstrates improvement on cardiomyocyte hypertrophy induced by ET1, which may be mediated by activating IP prostanoid receptor and the cyclic adenosine monophosphate–dependent signaling in cardiomyocytes. Shinmura et al. reported PGI2 analog. Alleviates oxidative injury of myocyte through opening mitochondrial ATP-sensitive K^+^ channels *via* the EP3 receptor (116). PGI2 analog showed protective effects on cardiac function through decreasing cardiomyocyte apoptosis.

### Neuregulin-1

NRG-1 derived from coronary endothelial cells, is a growth factor regulating the size, structure, and survival of cardiomyocytes. NRG-1 regulates downstream signal pathways by binding to the tyrosine kinase receptor erythroblastic leukemia viral oncogene homolog 4 on the cellular membranes of the myocytes. The activation of downstream pathways includes extracellular signal-regulated kinase 1/2 (ERK1/2) and phosphoinositide 3-kinase (PI3K)/AKT signaling pathways, which both take part in cardiomyocyte hypertrophy. In addition, NRG-1 is documented to improve cardiomyocyte apoptosis through an Akt-dependent pathway. Moreover, NRG and recombinant NRG-1β protect cardiomyocytes against the cardiomyocyte death induced by anthracycline, β-adrenergic receptor, and H_2_O_2_. Activated NRG-1/ErbB4 signaling or ErbB4 expression causes cardiomyocyte proliferation and promotes myocardial regeneration after cardiac injury (117).

### Apelin

Endothelium in heart secrets Apelin to oppose the effects of the rennin-angiotensin system. The expression of Apelin increases in response to hypoxia in the ischemic heart, further promoting vasodilation *via* nitric-oxide dependent mechanism. Moreover, Apelin regulates cardiomyocytes *via* binding its GPCR receptor APJ (118).

## Clinical Assessment of Endothelial Function and Strategy for Endothelial Protection

Endothelium function assessment includes evaluating the responsiveness of endothelial cells to the stimulus of vasodilation or vasoconstriction. With the ongoing understanding of endothelial dysfunction, the evaluation of endothelial function is considered as an important way of assessing disease development, with potential clinical applicability as a risk factor in cardiovascular complications, even in asymptomatic patients.

And invasive assessment is a direct method to evaluate vasodilation in response to endothelium-dependent and -independent stimuli. The occurrence and development of pathogenic events in cardiovascular complications are always accompanied by the alterations of circulating biomarkers, including inflammation-related cytokines (IL-1β and IL-6), cell surface induced adhesion molecules (E-selectin and VCAM-1), and systemic indicators of inflammation (high-sensitivity c-reactive protein), endothelin-1, NRG-1, and so on. The measurement of circulating biomarkers is a practicable way to assess endothelial function. Moreover, noninvasive evaluations including echocardiography, magnetic resonance imaging, and positron emission tomography also provide a potential risk of future cardiometabolic events, classified as low, medium, or high risk, which is conducive to the prevention and early treatment of diseases.

Furthermore, imaging technology is a relatively visualized way to evaluate the vascular injury and endothelial dysfunction (119). Currently, there are some methods to evaluate endothelial dysfunction, but endothelial dysfunction fails to meet diagnostic criteria or therapeutic targets. Furthermore, new noninvasive methods for assessing endothelial function from experimental studies to human patients are required, precise elevation of endothelial function would provide the possibility for endothelial dysfunction to become the standard of clinical diagnosis and treatment, further becoming a therapeutic target.

Currently available cardiovascular drugs, including angiotensin-converting enzyme (ACE) inhibitors, AT1 receptor antagonists, statins and many antioxidant and anti-inflammatory cardiovascular drugs, are therapeutic strategies. In addition, repair therapy with pharmacological agents, epigenetic approaches, endothelium-specific antioxidant and anti-inflammatory drug-delivery are promising candidates for the future. Moreover, exercise and dietary changes are highly potent “natural” alternatives (120).

## Conclusions and Future Perspectives

Patients with early diabetic cardiomyopathy are asymptomatic, which brings difficulties to early diagnosis and treatment (11). Abnormal myocardial metabolism including enhanced FA uptake and insulin signaling cascade has been present in these patients (121). With the progression of diabetic cardiomyopathy, there is a decrease in insulin secretion, leading to increased blood glucose levels and increased FA oxidation. However, excessive fatty acids are provided to cardiomyocytes, leading to the damage of cardiomyocytes (109). As diabetic cardiomyopathy is characterized by decreased capillary density, enhanced requirement for oxygen in FA mitochondrial metabolism presents a challenge to cardiac metabolism. Moreover, scarce oxygen delivery cannot match augmented FA uptake, leading to increased FA storage as TG in cardiomyocytes, which may induce cardiomyocyte death by lipotoxic damage (122).

Endothelial dysfunction has been regarded as a crucial link in diabetic cardiomyopathy. The role of endothelial dysfunction in the development of diabetic cardiomyopathy involves many aspects, including impaired NO activity, disturbed metabolism, aberrant ROS production, and oxidative stress. Direct evidence provided by scRNA-seq demonstrated the character of EC dysfunction *in vitro* from a single-cell transcriptome level. Substantial changes occur in ECs, including gene expression and phenotypic change, when exposed to chronic high glucose and inflammation. As endothelial dysfunction has been identified as a key factor in diabetic cardiomyopathy, it is urgent to evaluate the precise role of ECs in the development and progression of diabetic cardiomyopathy. On the one hand, endothelial dysfunction contributes to the development of diabetes and indirect cardiac function damage; on the other hand, endothelial dysfunction also leads to direct damage of heart. Moreover, ECs are heterogeneous across tissues, which remains further investigation. The single-cell transcriptome atlas of endothelial is important tools, which provide an access to explore the difference among ECs in diverse organs and the precise role of ECs from different vascular beds. Different phenotypes and percentages of ECs have been shown, LECs take part 3.7% in ECs in the heart, and LECs from hearts are major in LECs (123). In decades, a large amount of evidence has indicated the critical role of LECs in regulation of heart physiology and pathology (124–126). Lymphatic vessels not only regulate fluid drainage but also function as antigen-presenting. Currently, studies on the relationship between lymphatic vessels and diabetic cardiomyopathy are still lacking. Considering the important role of lymphatic vessels, the role of lymphatic vessels in DCM may become a new research direction in the future.

The crosstalk between ECs and cardiomyocytes is critical in cardiac development, and also plays a key role during the onset and progression of cardiac disease. ECs and cardiomyocytes are in close proximity and communicate through paracrine signals, as well as direct cell-to-cell contact (91). However, the specific transport molecules or interactions between ECs and cardiomyocytes during normal heart development and/or disease progression remain unclear. Further investigation is required to understand the crosstalk between endothelial-cardiomyocyte signals and cardiomyocyte-endothelial signals. Furthermore, it is necessary to establish more complex *in vitro* models to integrate ECs, cardiomyocytes, and other cell types into a three-dimensional structure, mimicking *in vivo* conditions.

The measurement of circulating biomarkers is another means to assess endothelial dysfunction. The occurrence and development of pathogenic events in cardiovascular complications are always accompanied by the alterations of circulating biomarkers, including inflammation-related cytokines (IL-1β and IL-6), cell surface induced adhesion molecules (E-selectin and VCAM-1), and systemic indicators of inflammation (high-sensitivity c-reactive protein), endothelin-1, NRG-1, von Willebrand factor, thrombospondin-1, endothelial miRNAs (miR-146a), and endothelial microparticles. Convenient and effective evaluation of endothelial function, promotes the widespread application of endothelial dysfunction evaluation in clinical practice, and timely monitoring of endothelial dysfunction and the progression of diabetic cardiomyopathy.

Current therapeutic strategies for diabetic cardiomyopathy mainly focus on decreasing blood glucose, ignorant of the continuous progress of cardiac remodeling and heart failure. Novel DM medications significantly improve the prognosis of diabetic cardiomyopathy, which may be one of the reasons for saving endothelial function. SGLT2i improves endothelial function by increasing NO production, endothelium-dependent vasodilation, reducing vascular inflammation and oxidative stress, and attenuating endothelial cell senescence. Importantly, the improvement of SGLT2i is associated with increased Flow-mediated dilation (FMD). Novel DM medications show better clinical outcomes, which suggests their multiple effects are critical. It is unclear about the further mechanism of SGLT2i, more studies are needed.

Taken together, with the deepening understanding of the role of endothelial dysfunction in diabetic cardiomyopathy and the increase of methods for assessing endothelial dysfunction, endothelial dysfunction may become a new target for the clinical treatment of DCM in the future. Clinical therapies need to consider the extent of endothelial damage. Considering the population with severe endothelial dysfunction are more prone to be influenced by endothelial function, it is urgent to identify this population and give clinical interventions for endothelial dysfunction.

## Author Contributions

MW prepared the initial draft of the manuscript. SL supervised the study. YL and JL edited the manuscript for the intellectual content. All authors contributed to the article and approved the submitted version.

## Funding

This work was supported by the National Natural Science Foundation of China to SL [81974032] and JL [82070396].

## Conflict of Interest

The authors declare that the research was conducted in the absence of any commercial or financial relationships that could be construed as a potential conflict of interest.

## Publisher’s Note

All claims expressed in this article are solely those of the authors and do not necessarily represent those of their affiliated organizations, or those of the publisher, the editors and the reviewers. Any product that may be evaluated in this article, or claim that may be made by its manufacturer, is not guaranteed or endorsed by the publisher.
